# Impact of the COVID-19 pandemic on the activity of the Radiological Emergency Department: the experience of the Maggiore della Carità Hospital in Novara

**DOI:** 10.1007/s10140-021-01928-z

**Published:** 2021-04-04

**Authors:** C. Garlisi, D. Licandro, A. Siani, S. Rodolfi, S. Pansini, L. I. Garcia Navarro, A. Carriero, G. C. Avanzi, L. M. Castello

**Affiliations:** 1 SCDU Radiodiagnostica - Ospedale Maggiore Della Carità, Novara, Italy; 2 SCDU Medicina E Chirurgia D’Accettazione E D’Urgenza - Ospedale Maggiore Della Carità, Novara, Italy

**Keywords:** Coronavirus, COVID-19, Radiology, Emergency room, Healthcare system

## Abstract

**Purpose:**

During the first peak of the COVID-19 pandemic, the activity of Emergency Departments worldwide changed dramatically, focusing on diagnosis and care of the Sars-Cov-2 associated disease. These major changes also involved the activity of the Emergency Radiology Department (ERD). This study aimed to analyse the impact of the COVID-19 pandemic on imaging studies, both in terms of the amount, frequency and subspecialty of different imaging modalities requested to the ERD of the Maggiore della Carità Hospital in Novara (Italy).

**Methods:**

To this end, our observational study took into account the imaging studies requested by the emergency department during three-time spans. These were defined as phase 0 (pre-pandemic), phase 1 (pandemic peak with complete lockdown) and phase 2 (post-pandemic peak with partial lifting of restrictive measures), as derived from Italian urgent decrees by the President of the Council of Ministers (DPCM) which established the duration and entity of the lockdown measures throughout the pandemic. The dataset was processed and then compared with Pearson’s chi-squared test.

**Results:**

During the pandemic peak, our data showed a significant drop in the total number of studies requested and a significant rise in computed tomography (CT) studies. In particular, a statistically significant increase in chest CT studies was found, probably due to the high sensitivity of this imaging method in identifying pulmonary involvement during respiratory tract infection of possible viral etiology (SARS-Cov-2). Moreover, we observed a statistically significant decrease of X-ray (XR) and ultrasound (US) studies during phase 1 compared to phase 0 and phase 2 probably due to a reduction in the numbers of ER visits for minor traumas given the mobility restrictions and people hesitancy in visiting the ER due to fear of contagion.

**Conclusions:**

We can conclude that the activity of the ERD was heavily impacted by the SARS-Cov-2 pandemic. Further studies will be needed to estimate the impact of the pandemic on public health in terms of excess mortality related to delayed diagnosis and care of non-COVID diseases.

## Introduction

Between the end of February and the beginning of March 2020, the Sars-nCov-2 pandemic reshaped the way hospitals and their radiology departments provide for healthcare needs of the population, both nationally and locally. Meanwhile, emergency rooms faced the greatest challenge as the general population tended to reduce its visits to the emergency room and suspected COVID-19 patients started pouring in [1]. This is probably partly due to state-mandated lockdowns which greatly reduced transport-related accidents and minor traumas during physical activities, and partly since citizens actively avoided visits to the ER to protect themselves from the risk of infection. This might have led to a worsening of the general health of people suffering from other chronic and acute diseases and conditions (cardiovascular, diabetic, oncological, etc.…) [2]. The University Hospital “Maggiore della Carità” of Novara is the main trauma centre in the north-eastern part of the region of Piedmont, and according to its website (http://www.maggioreosp.novara.it/lospedale-maggiore/chi-siamo/lazienda-di-oggi/), visits to its Emergency Department were 71,730 in 2019. During the COVID-19 pandemic, the whole province of Novara was significantly affected. Being at the border with Lombardy, the most affected region in Italy so far. Until 24/06/2020, the province of Novara had 2780 confirmed COVID-19 cases out of a total of 31,276 cases for the Piedmont region (http://www.salute.gov.it/imgs/C_17_notizie_4933_1_file.pdf). During the pandemic, the activity of the whole Radiology department was necessarily reshaped, in line with regional and national provisions, and in the same way as other main hospitals, such as in Milan [3]. Restrictions were put on the healthcare services to citizens, suspending deferrable and non-urgent hospitalisation and outpatient activities, in order to minimize unnecessary contacts between staff and patients, and among the patients themselves. It is estimated that 70% of the Italian imaging providers have focused their activity on emergency studies and procedures [4]. Conversely, the rostering of the ERD reflected this, to accommodate the increasing demand for chest CTs, which have been widely used as a crucial diagnostic and prognostic tool in patients with both suspected and confirmed COVID-19 [5]. For instance, medical and technical staff presence in the ERD was doubled at all times. In particular, two consultant radiologists and two residents were rostered each shift, the latter reading studies remotely, in order to allow proper distancing.

Furthermore, in order to reduce the spread of the disease among patients and healthcare professionals, hospital management rolled out and frequently updated operational guidelines, usually inspired by WHO guidelines and the latest scientific evidence.

## Purpose

During the first wave of the pandemic, we observed a significant drop in the total amount of studies requested to our institution’s ERD. In those difficult days, our ER-diagnostic activity has mainly focused on COVID-19, though at the same time it was necessary to provide all the necessary diagnostic effort to surgical and medical major emergencies that continued accessing our ER. The aim of this study was to evaluate the impact of the COVID-19 pandemic in shaping the activity of our ERD in terms of studies performed, and to be a starting point for further studies aimed at highlighting the impact on both acute and chronic healthcare provision and relative treatment outcomes during the pandemic.

## Methods

Data regarding the imaging studies performed in the Emergency Radiology of the AOU Maggiore della Carità were obtained from Fenix, our radiology information system (**RIS**), and exported in a database created with Microsoft Excel. Only imaging studies which met the following inclusion criteria were considered:

- Studies performed in the Emergency Radiology Department
- Studies performed from 12/01/2020 to 30/06/2020
- Studies requested from the E.D. physician for patients accessing the E.D.

We excluded all other studies performed by ERD staff, such as portable x-rays and Out of Hours studies requested for patients already admitted.

Data collected included the type of exam performed and the outcome of the exam in relation to the diagnostic query of the requesting clinician [6].

The data collected was divided into three timeframes of the same extent, named phase 0, phase 1 and phase 2:

- Phase 0: from 12/01/20 to 08/03/20
- Phase 1: from 9/03/20 to 04/05/20
- Phase 2: from 05/05/20 to 30/06/20

This partition was agreed on according to the Italian contagion curve of the COVID-19 pandemic as proposed by Worldometers [7], which indicates that the peak amount of new cases was reached on 21/03/2020 in our country. We also took into account the urgent decrees by the Italian Council of Ministers. These included *DCPM 9*/03/*2020*, which marked the beginning of the Italian “lockdown” and *DCPM 26*/04/*2020*, which eased some of the freedom of movement restrictions previously introduced and marked the beginning of the so-called phase 2 in our country.

From our database, we extracted the relative frequencies of each type of study pertaining to our timeframes and the amount of studies that were congruent compared to the clinician’s query. Pivot tables were then built in order to compare the relative frequencies observed of requested studies during the first COVID-19 outbreak with those observed before and after (phase 0 and phase 2). Pearson’s chi-squared test was performed in order to assess the statistical significance of the differences in frequency distribution observed. Significance value was set at *p* < 0.05.

## Results

Relative frequencies and percentages of each type of study request divided by timespan are reported in Table 1.

**Table1:** Summary of frequency distributions of the imaging studies requested to our emergency radiology service

	Phase 0		Phase 1		Phase 2	
	*n*	%	*n*	%	*n*	%
Conventional radiology						
No XR	2193	26.82	2465	59.40	2559	43.79
Skeletal XR	3002	36.71	860	20.72	1917	32.80
Chest XR	2437	29.80	647	15.59	1038	17.76
Abdomen XR	546	6.68	178	4.29	330	5.65
Total requests	8178		4150		5844	
CT scans						
No CT scan	6446	78.82	1800	43.37	3525	60.32
Head CT scan	992	12.13	486	11.71	836	14.31
Chest CT scan	169	2.07	1529	36.84	833	14.25
Abdomen CT scan	281	3.44	203	4.89	376	6.43
Facial bones CT	105	1.28	63	1.52	131	2.24
Spine CT scan	142	1.74	46	1.11	103	1.76
Limbs CT scan	37	0.45	16	0.39	31	0.53
Neck CT scan	6	0.07	7	0.17	9	0.15
Total requests	8178		4150		5844	
Ultrasound						
No US	7718	94.38	4041	97.37	5604	95.89
Muscoloskeletal US	4	0.05	1	0.02	1	0.02
Upper abdomen US	32	0.39	7	0.17	1	0.02
Lower abdomen US	2	0.02	2	0.05	2	0.03
Abdomino-pelvic US	422	5.16	99	2.39	236	4.04
Total requests	8178		4150		5844	
Positive or negative						
Positive	2190	26.78	1543	37.18	1536	26.28
Negative	5988	73.22	2607	62.82	4308	73.72
Total requests	8178		4150		5844	

The differences observed between the relative frequencies of phase 0 and phase 1 were statistically significant (*p* < 0.0001) for conventional radiology studies, for CT scans, for US studies and also in terms of studies with findings congruent with the clinician’s query studies.

In particular, during phase 1 compared to phase 0, a significant decrease not only in skeletal X-ray (*n*_0_ = 3002 (36.71%) vs *n*_1_ = 860 (20.72%)) but also in the number of chest (*n*_0_ = 2437 (29.80%) vs *n*_1_ = 647 (15.59%)) and abdomen X-ray (*n*_0_ = 546 (6.68%) vs *n*_1_ = 178 (4.29%)) was observed. As expected, a significant increase in chest CT (*n*_0_ = 169 (2.07%) vs *n*_1_ = 1529 (36.84%)) was noticed as a result of the huge increase in patients with respiratory complaints reaching the ER department.

On the other hand, other types of CT studies showed no significant difference in frequency between phase 0 and phase 1 frequencies values; only a non-significant reduction in head CT scans was identified (*n*_0_ = 992 (12.13%) vs *n*_1_ = 486 (11.71%)).

During phase 1, we observed a significant reduction of all types of US studies.

The percentage of studies congruent with the clinician’s query were significantly higher (*p* < 0.0001) during phase 1 (37.18%) than during phase 0 (26.78%) whereas the amount of negative studies decreased during phase 1 (62.82%) and was higher in phase 0 (73.22%) (Table 2).

**Table 2:** Comparison between phase 1 and phase 0

	Phase 0		Phase 1	
Conventional radiology				
No XR	2193		2465	
Skeletal XR	3002		860	
Chest XR	2437		647	
Abdomen XR	546		178	*p* < 0.0001
CT scans				
No CT scan	6446		1800	
Head CT scan	992		486	
Chest CT scan	169		1529	
Abdomen CT scan	281		203	
Facial bones CT	105		63	
Spine CT scan	142		46	
Limbs CT scan	37		16	
Neck CT scan	6		7	*p* < 0.0001
Ultrasound studies				
No US	7718	4041		
Muscoloskeletal US	4	1		
Upper abdomen US	32	7		
Lower abdomen US	2	2		
Abdomino-pelvic US	422	99		*p* < 0.0001
Positive vs negative				
Positive	2190	1543		
Negative	5988	2607		*p* < 0.0001

Comparing the relative frequencies of phase 1 to phase 2, significant differences (*p* < 0.0001) were observed for conventional radiology, CT scans and for US studies.

In regard to the conventional radiology studies, the most striking difference was observed for skeletal X-ray (XR) in terms of expected values with a raise in skeletal X-ray studies during phase 2 following a drop of requests during phase 1 (*n*_1_ = 860 (20.72%) vs *n*_2_ = 1917 (32.80%)).

CT scans as a whole showed significant frequency differences between phases, mainly sustained by the decrease in chest CT scan requests (*n*_1_ =1529 (36.84%) vs *n*_2_ = 833 (14.25%)) and a slight increase in head CT scans during phase 2 (*n*_1_ = 486 (11.71%) vs *n*_2_ = 836 (14.31%)).

Abdomino-pelvic US studies showed significant differences in terms of frequency distribution with a significant decrease during phase 1 versus phase 0 (*n*_0_ = 422 vs *n*_1_ = 99) followed by a significant increase in phase 2 versus phase 2 (*n*_1_ = 99 vs *n*_2_ = 236).

The percentage of studies congruent with the clinician’s query were significantly higher during phase 1 (37.18%) than during phase 2 (26.28%) (Table 3).

**Table 3:** Comparison between phase 2 and phase 1

	Phase 2	Phase 1	
Conventional radiology			
No XR	2559	2465	
Skeletal XR	1917	860	
Chest XR	1038	647	
Abdomen XR	330	178	*p* < 0.0001
CT scans			
No CT scan	3525	1800	
Head CT scan	836	486	
Chest CT scan	833	1529	
Abdomen CT scan	376	203	
Facial bones CT	131	63	
Spine CT scan	103	46	
Limbs CT scan	31	16	
Neck CT scan	9	7	*p* < 0.0001
Ultrasound studies			
No US	5604	4041	
Muscoloskeletal US	1	1	
Upper abdomen US	1	7	
Lower abdomen US	2	2	
Abdomino-pelvic US	236	99	*p* < 0.0001
Positive vs negative			
Positive	1536	1543	
Negative	4308	2607	*p* < 0.0001

Finally, a comparison between phase 0 and phase 2 studies was made, showing again significant differences for conventional radiology (*p* < 0.0001), CT scans (*p* < 0.0001) and US studies (*p* < 0.000003).

In particular, the number of chest X-rays performed decreased significantly during phase 2 in respect to phase 0, when it was significantly higher (*n*_0_ = 2437 vs *n*_2_ = 1038). Moreover, the total number of other studies (“no X-rays”) was significantly higher during phase 2 than during phase 0 (*n*_0_ = 2193 vs *n*_2_ = 2559).

CT scans as a whole showed significant differences between the two phases mainly sustained by the increase in chest CT requests during phase 2 compared to phase 0 (*n*_0_ = 169 vs *n*_2_ = 833) and by a slight increase in abdomen CT scans in phase 2 (*n*_0_ = 281 vs *n*_2_ = 376).

Lastly, during phase 2, the abdomino-pelvic US and the upper abdomen US studies were significantly less performed compared to phase 0 (*n*_0_ = 422 vs *n*_2_ = 236).

No statistically significant difference (*p* > 0.05) was found between the amount of studies congruent with the clinician’s query between phase 0 (26.78%) and phase 2 (26.28%) (Table 4).

**Table 4:** Comparison between phase 2 and phase 0

	Phase 2	Phase 0	
Conventional radiology			
No XR	2559	2193	
Skeletal XR	1917	3002	
Chest XR	1038	2437	
Abdomen XR	330	546	*p* < 0.0001
CT scans			
No CT scan	3525	6446	
Head CT scan	836	992	
Chest CT scan	833	169	
Abdomen CT scan	376	281	
Facial bones CT	131	105	
Spine CT scan	103	142	
Limbs CT scan	31	37	
Neck CT scan	9	6	*p* < 0.0001
Ultrasound studies			
No US	5604	7718	
Muscoloskeletal US	1	4	
Upper abdomen US	1	32	
Lower abdomen US	2	2	
Abdomino-pelvic US	236	99	*p* < 0.0001
Positive vs negative			
Positive	1536	2190	
Negative	4308	5988	*p* > 0.05

## Discussion

As our results show, a statistically significant decrease in the frequency of conventional radiology studies was observed during the COVID-19 outbreak. The greatest frequency reduction was observed for chest X-ray requests (the number of CXRs performed was 2437 in phase 0, 647 in phase 1 and 1038 in phase 2 with statistically significant differences observed both between phase 0 and phase 1 and between phase 1 and phase 2). At the same time, we observed a huge increase in the number of CT scans performed during phase 1, compared to phases 0 and 2: in spite of many guidelines speaking against CT scan as the first line study in diagnosing COVID-19, due to its lower sensitivity (80–90%) than the RT-PCR nasopharyngeal swab (95–97%) [8], our centre (as many others) widely employed it in any patient with acute respiratory symptoms during the COVID-19 outbreak. According to a large multicentre study by the IAEA, most of the centres involved did not use CT for the first diagnosis, but possibly as a tool for assessing the severity of lung involvement. The use of CT at the patient’s entry point would be justified in a situation of high prevalence of suspicious clinical presentations and a lack of means to timely obtain RT-PCR swabs results [5]. This tendency was still apparent during phase 2, when the amount of chest CT scans failed to set back to the pre-COVID-19 levels as shown by the statistically significant differences observed in the respective frequency distribution between phase 0 and phase 2.

**Fig. 1:**
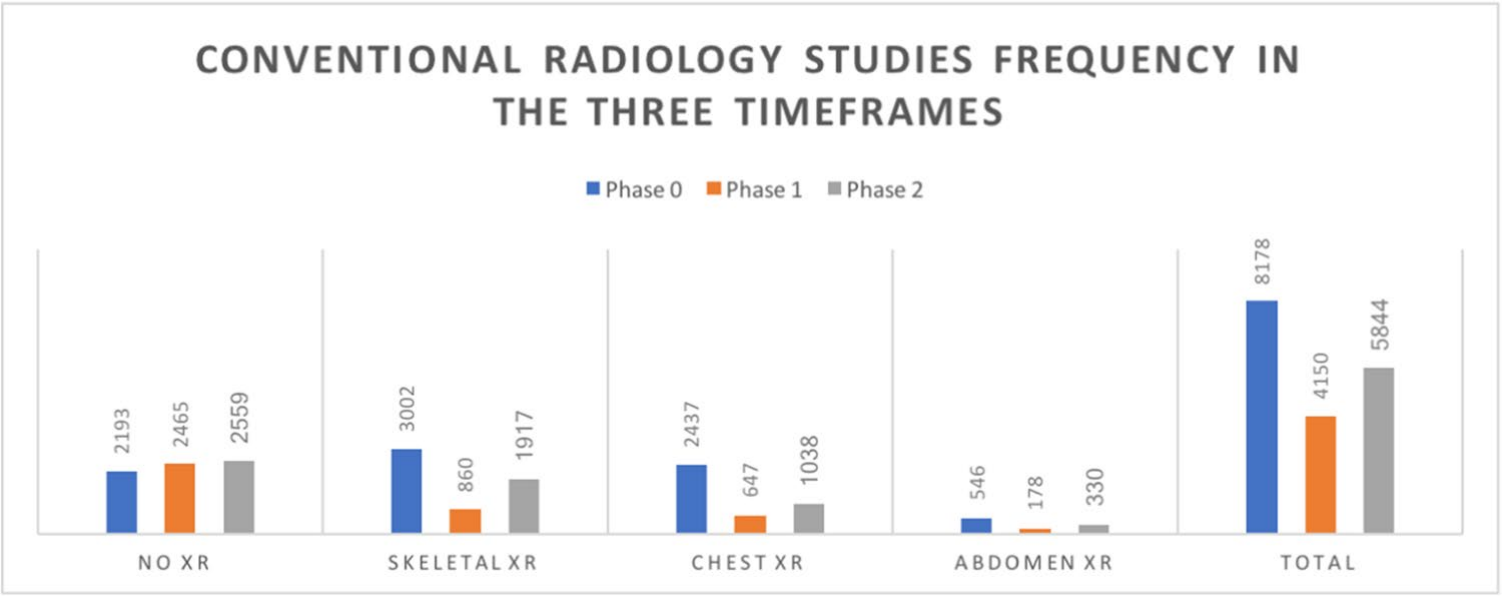
Frequency distribution of conventional radiology exams in the three timeframes

**Fig. 2:**
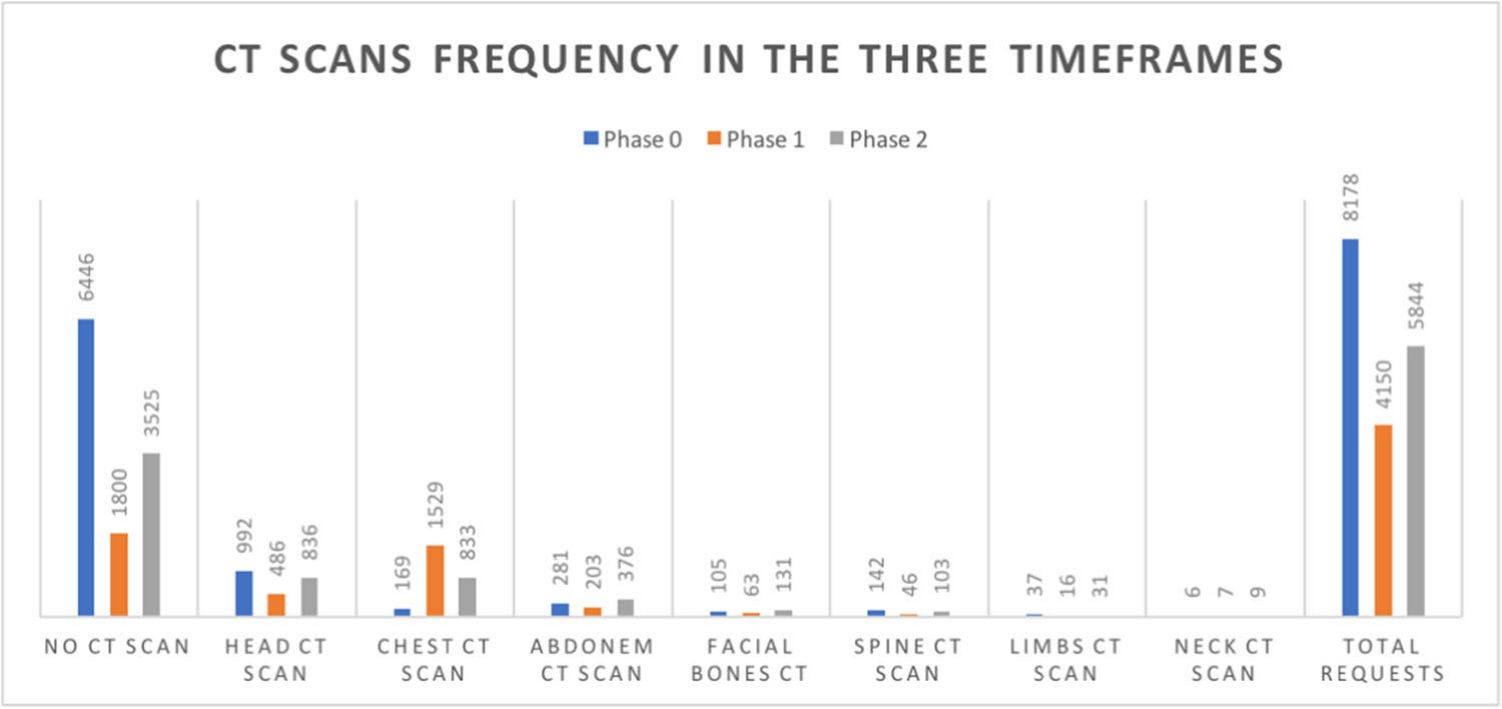
Frequency distribution of CT scans in the three timeframes

A significant reduction of the number of MSK X-ray studies was also identified during the COVID-19 outbreak. In particular, the percentage of such studies dropped from 36.71% of overall studies in phase 0 to 20.71% during phase 1 and failed to return to the baseline during phase 2 (32.8%). Variations in the frequency of conventional radiology studies are summarized by Fig. 1. Also, a decrease in the number of abdomen ultrasound studies performed in the ER was observed in phase 1, and even in phase 2 the number of abdomen ultrasound studies failed to regain phase 0 frequencies. Interestingly, the frequency of head CT scans showed no significant difference among the three timeframes (Fig. 2).

**Fig. 3:**
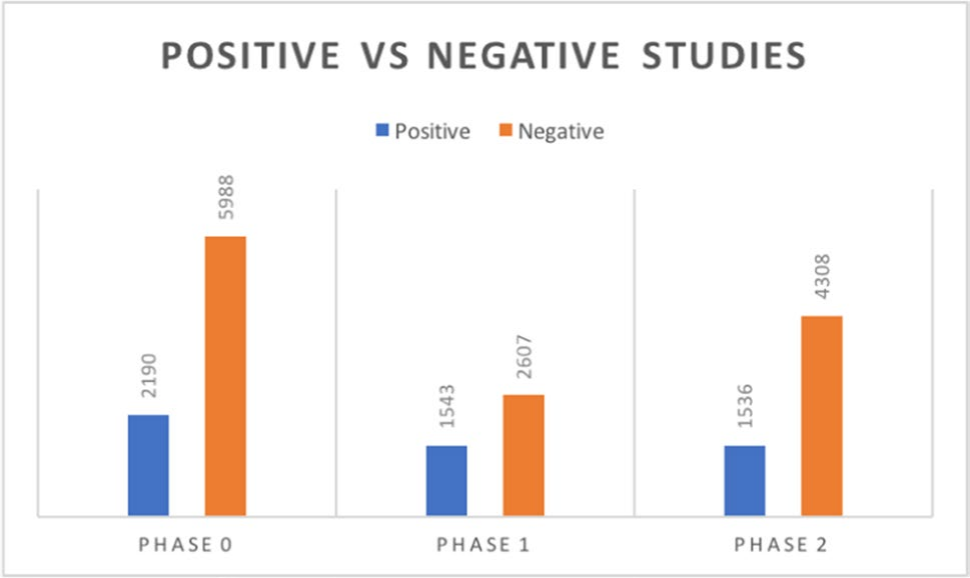
Amount of positive and negative exams for every timeframe

Last but not least, data about the amount of studies that were congruent with the clinician’s query turned out to be statistically significant: the percentage of studies congruent with the clinician’s query turned out to be 37.18% during phase 1, while it was 26.78% in phase 0 and 26.28% in phase 2. No statistically significant difference was found between the amount of studies congruent with the clinician’s query in phase 0 and in phase 2, suggesting that this parameter went back to normal after the acute phase of the outbreak (Fig. 3). In general, the emergency radiological activity decreased by about 50% in the acute phase of the pandemic (phase 1) to rise again, in the post-lockdown phase (phase 2), to about 71% of the pre-pandemic phase (phase 0).

It is reasonable to think that the increase in the amount of studies congruent with the clinician’s query during phase 1 may have been due to the huge number of chest CT scans performed during the COVID-19 outbreak, when in our area there was a high prevalence of Sars-nCov-2 infections and that a more frequent use of chest CT may have contributed to reduce the number of chest X-rays performed. It is not completely clear why the frequency of MSK X-ray studies and of ultrasound scans was so sharply reduced during phase 1. This is possibly partly related to the intrinsic limitation of our study which could not include full data from electronic patient records for hurdles in data acquisition and analysis which led to quite inhomogeneous raw data.

Although our results are partly expected and unsurprising, there are some questions that remain unanswered. Further research is needed to understand whether those differences were associated with a different frequency of presentation for ailments managed in the emergency room or with differences in patients’ management due to the emergency. For this purpose, it would be of some interest to investigate if and how the clinician’s rationale for ordering studies changed during the COVID19 outbreak.

The main limitation of our study is our inability to normalise our dataset by comparing it with analogous data from the same months of 2019. This does not allow us to take into account seasonal variation in ER visits in our analysis.

Furthermore, it would be interesting to analyse data on TATs from image acquisition to image reading. Unfortunately, this aspect has not been included in our study.

## Conclusions

The SARS-nCov-2 pandemic led to an overall decrease in the amount of imaging studies in the emergency setting. At the same time, we detected an increase in chest CTs, in relation to the greater diagnostic sensitivity compared to chest x-ray in ruling out interstitial pneumonia.

The overall rate of studies congruent with the clinician’s query increased significantly during the acute phase of the pandemic (phase 1), likely due to high amount of chest CT scans positive for interstitial pneumonia in that timeframe, coupled with the sharp increase of chest CT requests.

This reduction in the overall number of studies in the ERD, with the notable exception of chest CTs, might have been due to the public’s hesitancy in accessing the ER. Hospitals might have been perceived as a very dangerous place in terms of the risk of contracting the Sars-nCov-2 infection. It could be interesting to further elaborate on this theme by administering a questionnaire to a representative sample of the general population relating to their feelings regarding visits to the hospital during the different phases of the pandemic and during the same timeframe of 2019.

## Data Availability

Not applicable.
